# Levosimendan affects oxidative and inflammatory pathways in the diaphragm of ventilated endotoxemic mice

**DOI:** 10.1186/s13054-015-0798-8

**Published:** 2015-03-02

**Authors:** Willem-Jan M Schellekens, Hieronymus WH van Hees, Marianne Linkels, PN Richard Dekhuijzen, Gert Jan Scheffer, Johannes G van der Hoeven, Leo MA Heunks

**Affiliations:** Department of Anesthesiology, Radboud University Medical Centre, Postbox 9101, Nijmegen, 6500 HB the Netherlands; Department of Pulmonary Diseases, Radboud University Medical Centre, Postbox 9101, Nijmegen, 6500 HB the Netherlands; Department of Intensive Care Medicine, Radboud University Medical Centre, Postbox 9101, Nijmegen, 6500 HB the Netherlands

## Abstract

**Introduction:**

Controlled mechanical ventilation and endotoxemia are associated with diaphragm muscle atrophy and dysfunction. Oxidative stress and activation of inflammatory pathways are involved in the pathogenesis of diaphragmatic dysfunction. Levosimendan, a cardiac inotrope, has been reported to possess anti-oxidative and anti-inflammatory properties. The aim of the present study was to investigate the effects of levosimendan on markers for diaphragm nitrosative and oxidative stress, inflammation and proteolysis in a mouse model of endotoxemia and mechanical ventilation.

**Methods:**

Three groups were studied: (1) unventilated mice (CON, n =8), (2) mechanically ventilated endotoxemic mice (MV LPS, n =17) and (3) mechanically ventilated endotoxemic mice treated with levosimendan (MV LPS + L, n =17). Immediately after anesthesia (CON) or after 8 hours of mechanical ventilation, blood and diaphragm muscle were harvested for biochemical analysis.

**Results:**

Mechanical ventilation and endotoxemia increased expression of inducible nitric oxide synthase (iNOS) mRNA and cytokine levels of interleukin (IL)-1β, IL-6 and keratinocyte-derived chemokine, and decreased IL-10, in the diaphragm; however, they had no effect on protein nitrosylation and 4-hydroxy-2-nonenal protein concentrations. Levosimendan decreased nitrosylated proteins by 10% (*P* <0.05) and 4-hydroxy-2-nonenal protein concentrations by 13% (*P* <0.05), but it augmented the rise of iNOS mRNA by 47% (*P* <0.05). Levosimendan did not affect the inflammatory response in the diaphragm induced by mechanical ventilation and endotoxemia.

**Conclusions:**

Mechanical ventilation in combination with endotoxemia results in systemic and diaphragmatic inflammation. Levosimendan partly decreased markers of nitrosative and oxidative stress, but did not affect the inflammatory response.

## Introduction

Respiratory muscle dysfunction frequently develops in critically ill patients [1-4]. Clinical entities associated with respiratory muscle dysfunction in these patients include systemic inflammation [3,5] and controlled mechanical ventilation (MV) [6]. Recent data obtained from both animal and human studies have provided insight into the biochemical pathways underlying muscle atrophy and dysfunction in these patients [1,7-9]. Nitrosative and oxidative protein modifications are increased in the diaphragm during both MV and experimental sepsis [10,11]. Moreover, protein degradation is increased in the diaphragm in mechanically ventilated animals and patients [2,12,13]. Inflammatory cytokines are well-known modulators of muscle protein turnover during endotoxemia [14,15] and are increased in the diaphragm after MV [16,17]. For example, tumor necrosis factor (TNF)-α activates the proteolytic enzymes caspase-8 and caspase-3 in the diaphragm of mice exposed to lipopolysaccharide (LPS) [5]. Accordingly, strategies that reduce oxidative stress or inflammation may limit respiratory muscle dysfunction due to endotoxemia and MV [11,17].

Levosimendan is a cardiac inotrope approved for the treatment of heart failure in many countries worldwide. We have recently shown that levosimendan also improves respiratory muscle function in healthy subjects and patients with chronic obstructive pulmonary disease (COPD) [18,19]. The mechanisms of levosimendan include calcium sensitization of the contractile proteins and vasodilation through activation of ATP-sensitive potassium channels [20]. Interestingly, previous studies have shown that levosimendan reduces inflammation as well as oxidative and nitrosative stress [21-23]. For instance, in experimental septic rodents, levosimendan decreases interleukin (IL)-1β levels [24]. In addition, levosimendan decreases LPS-induced upregulation of IL-6, nitrite production and inducible nitric oxide synthase (iNOS) protein expression in macrophages and fibroblasts [21]. However, the effects of levosimendan on inflammation and oxidative and nitrosative stress in muscle have not been investigated, despite the importance of these pathways in intensive care unit (ICU)-acquired muscle weakness.

Accordingly, the hypothesis of the present study is that levosimendan dampens both oxidative and nitrosative stress and the inflammatory response in the diaphragm induced by endotoxemia.

## Methods

### Animals

Experiments were performed using male C57BL/6 mice aged 19 ± 0.5 weeks and body weight 24 ± 0.3 g (Charles River Laboratories, Sulzfeld, Germany).

To test the hypothesis that levosimendan dampens both oxidative and nitrosative stress and the inflammatory response in the diaphragm induced by MV and experimental sepsis, 42 mice were divided into 3 groups: (1) unventilated controls (CON, n =8), (2) MV and LPS (MV LPS, n =17) and (3) MV and LPS + levosimendan (MV LPS + L), n =17). The effects of MV on inflammatory markers in mice have been investigated previously in our laboratory [16]. Because patients admitted to the ICU with endotoxemia often require MV, we used an animal model in which endotoxemia was administered prior to MV. In both groups, LPS (10 μg) was administered by intraperitoneal injection immediately before initiation of MV.

The final choice for the dose of LPS was based on pilot experiments. In a pilot study, we found that administration of 100 μg of LPS resulted in high mortality. Nevertheless, immediately after intraperitoneal injection of a LPS bolus, one mouse in the MV LPS + L group died and was excluded from the study. At the start of MV, mice in the levosimendan group received levosimendan (Orion Pharma, Espoo, Finland) via the tail vein at a dose of 24 μg/kg body weight in 5% glucose solution. Subsequently, levosimendan (0.2 μg/kg/min intravenously) was administered during MV [20]. The experiments were approved by the regional animal ethics committee (Nijmegen, the Netherlands) and performed under the guidelines of the Dutch Council for Animal Care.

### Controlled mechanical ventilation

Mice selected for MV were anesthetized and mechanically ventilated as described previously [16]. Briefly, mice were anesthetized with an intraperitoneal injection of a combination of ketamine, medetomidine, and atropine (KMA): 7.5 μl/g body weight of induction KMA mix (consisting of 1.26 ml of ketamine, 100 mg/ml; 0.2 ml of medetomidine, 1 mg/ml; 1 ml of atropine, 0.5 mg/ml; and 5 ml of NaCl, 0.9%). Tidal volume was 8 ml/kg body weight, respiratory rate was 170/min, positive end-expiratory pressure was 1.5 cmH_2_O and inspired oxygen fraction was 0.45. A sterile catheter was inserted into the carotid artery for continuous blood pressure measurement and blood sampling at the end of the experiment (i-STAT blood chemistry analyzer; Abbott, Hoofddorp, the Netherlands). To maintain anesthesia, a 5.0 μl/g body weight bolus of KMA mix (consisting of 0.72 ml of ketamine, 100 mg/ml; 0.08 ml of medetomidine, 1 mg/ml; 0.3 ml of atropine, 0.5 mg/ml; and 18.9 ml of NaCl, 0.9%) was administered through an intraperitoneal catheter every 30 minutes. The CON mice were anesthetized and killed without being mechanically ventilated, as described previously [16].

### Tissue collection

Immediately after anesthesia (CON group) or after 8 hours of MV (MV LPS and MV LPS + L groups), mice were exsanguinated and a combined thoracotomy and laparotomy was performed. Left and right hemidiaphragm tissues were rinsed, quickly frozen in liquid nitrogen and stored at −80°C for later biochemical analysis [16].

### Nitrotyrosine

Protein nitrosylation was evaluated by detection of nitrotyrosine residues by performing SDS-PAGE, as described previously [25]. In short, diaphragm muscle was homogenized in ice-cold buffer (pH 7.2, 10 mM Tris-maleate, 3 mM ethylene glycol tetraacetic acid (EGTA), 275 mM sucrose, 0.1 mM dithiothreitol (DTT), 2 mg/ml leupeptin, 2 mg/ml aprotinin, 10 mg/L pepstatin A, 0.57 mM phenylmethylsulfonyl fluoride) using a Kinematica Polytron homogenizer (Kinematica, Lucerne, Switzerland), followed by three cycles of freeze-thawing and 30 minutes of centrifugation at 17,000 *g* at 4°C. Supernatant protein content was measured by Bradford analysis, and 20 μg of proteins in Laemmli buffer were analyzed according to standard Western blotting protocols. Blots were stained using an anti-nitrotyrosine antibody (clone 1A6; Upstate Biotechnology, Lake Placid, NY, USA) and goat anti-mouse IRDye 800CW (LI-COR Biosciences, Lincoln, NE, USA). Analysis was done using an Odyssey scanner and Odyssey 2.1 software (LI-COR Biosciences).

### Inducible nitric oxide synthase expression

mRNA levels of iNOS were determined by quantitative PCR (Mm01309902_m1 mouse assay; Applied Biosystems, Foster City, CA, USA) as described previously [26].

### 4-Hydroxy-2-nonenal protein

4-Hydroxy-2-nonenal (HNE) protein was analyzed as follows. Tissue samples were homogenized in 100 volumes of buffer (20 mM Tris, pH 7.4, 20 mM EGTA, 1 mM DTT, 0.5% SDS, 1 μl/20 mg protease inhibitor cocktail (P8340; Sigma-Aldrich Chemie, Zwijndrecht, The Netherlands)) in the Kinematica Polytron homogenizer. Samples were diluted in standard Laemmli sample buffer and boiled for 2 minutes. Ten microliters of each sample were analyzed according to standard Western blotting protocols. The following antibodies were used: anti-HNE (fluorophore rabbit polyclonal antibody, catalog number 393206; Calbiochem, Darmstadt, Germany), anti-actin (A2066; Sigma-Aldrich, St Louis, MO, USA) and IRDye 800CW goat anti-rabbit immunoglobulin G secondary antibody (926-32211; LI-COR Biosciences). An Odyssey scanner and Odyssey application software version 2.1 (LI-COR Biosciences) were used for analysis of HNE protein signaling.

### Cytokines in diaphragm and plasma

Levels of IL-1β, IL-10, TNF-α, IL-6 and keratinocyte-derived chemokine (KC) in the diaphragm and IL-1β, IL-10, TNF-α, IL-6 and KC in plasma were analyzed by enzyme-linked immunosorbent assay as described previously [27]. To determine cytokine levels in the diaphragm, the muscle was homogenized in 100 volumes of ice-cold buffer, pH 7.2 (10 mM Tris-maleate, 3 mM EGTA, 275 mM sucrose, 0.1 mM DTT, 2 mg/ml leupeptin, 2 mg/ml aprotinin, 10 mg/L pepstatin A, 0.57 mM phenylmethylsulfonyl fluoride), subjected to three cycles of freezing and thawing and centrifuged at 17,000 *g* at 4°C for 30 minutes [16]. Lower detection limits were 40 pg/ml for IL-10 and IL-1β; 32 pg/ml for TNF-α; and 160 pg/ml for IL-6 and KC.

### Caspase-3 activity

To assess involvement of proteolysis, we measured caspase-3 activity as described previously [26]. By using spectrophotometry, we determined the caspase-3 activity by measuring the generation of the fluorogenic cleavage product methylcoumarylamide from the fluorogenic substrate *N*-acetyl-Asp-Glu-Val-Asp-7-amido-4-methylcoumarin.

### MuRF1 and MAFbx expression

mRNA levels of muscle RING finger (MuRF1) and muscle atrophy factor box (MAFbx) were determined by quantitative PCR [26]. Levels of MuRF1 and MAFbx mRNA were normalized to glyceraldehyde 3-phosphate dehydrogenase (GAPDH) mRNA. The forward and reverse oligonucleotides used were, respectively, as follows: MuRF1: 5′-CAACCTGTGCCGCAAGTG-3′ and 5′-CAACCTCGTGCCTACAAGATG-3′; MAFbx: 5′-GACTGGACTTCTCGACTGCC-3′ and 5′-TCAGCCTCTGCATGATGTTC-3′; and GAPDH: 5′-TGATGGGTGTGAACCACGAG-3′ and 5′-GGGCCATCCACAGTCTTCTG-3′.

### Statistical analysis

A log-rank test was performed to test differences in survival between the MV LPS and MV LPS + L groups. Differences between groups regarding the time course of mean arterial pressure were evaluated with two-way analysis of variance (ANOVA). The D’Agostino-Pearson test was used to verify normal distribution of all parameters studied. An unpaired Student’s *t*-test was performed to evaluate the statistical significance of differences of the following normally distributed parameters (nitrosylated proteins and HNE protein between MV LPS and MV LPS + L animals). Differences in normally distributed parameters of diaphragm KC, plasma IL-10, IL-6, KC and caspase-3 activity were analyzed with one-way ANOVA. The Student-Newman-Keuls *post hoc* test was used to test the probability level of differences between nominal divided groups. Differences between parameters not normally distributed (iNOS mRNA; diaphragm IL-1β, IL-10, TNF-α, IL-6, plasma IL-1β and TNF-α; MuRF1 and MAFbx mRNA) were analyzed with one-way ANOVA and Kruskal-Wallis and Dunn’s *post hoc* tests. For statistical analysis of cytokine measurements, the value of the detection limit was used for samples that did not reach the detection limit. GraphPad Prism software was used to conduct statistical analysis (GraphPad Software, La Jolla, CA, USA). A probability level of <0.05 was considered significant. All data are presented as mean ± SD.

## Results

### Animal characteristics

**Figure 1:**
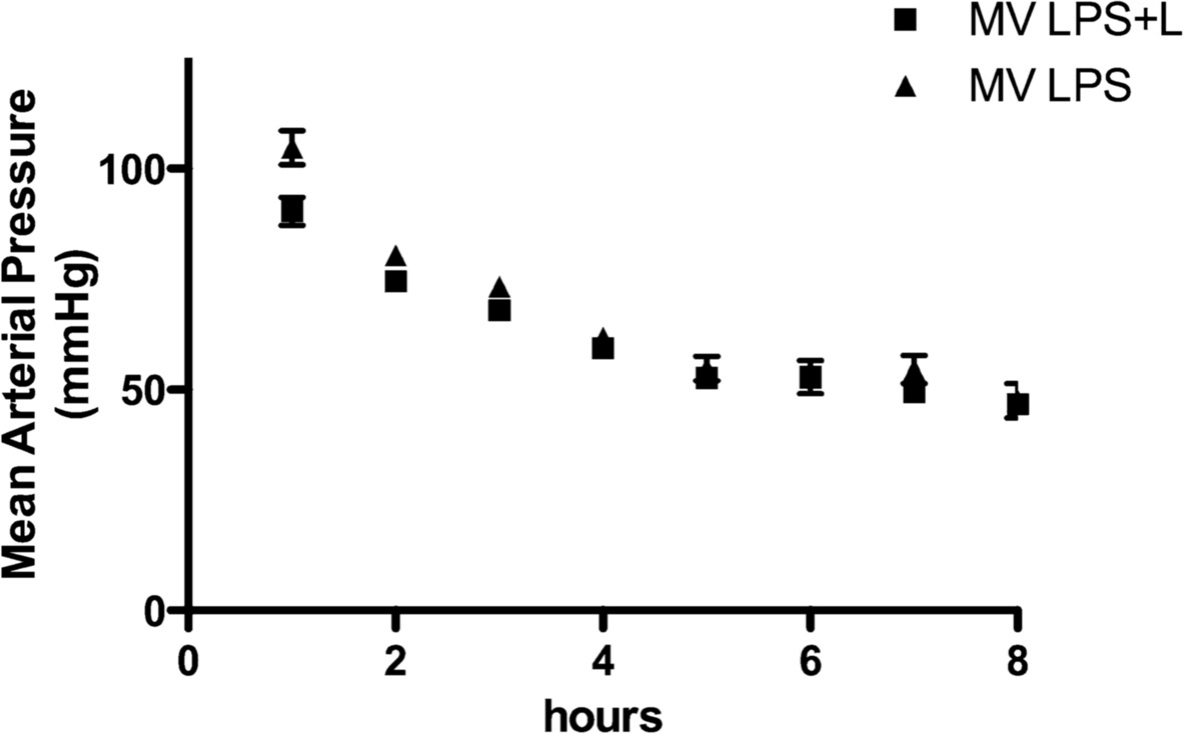
**Mean arterial pressure during 8 hours of mechanical ventilation.** LPS, Lipopolysaccharide; MV LPS, Mechanically ventilated endotoxemic mice; MV LPS + L, Mechanically ventilated endotoxemic mice treated with levosimendan.

Blood pressure decreased progressively in both groups during 8 hours of MV (Figure 1) (*P* =0.09), despite volume therapy (0.3 ml/hr). Blood gas analysis results at the end of the experiments are shown in Table 1. The alveolar-arterial (A-a) oxygen gradient was high after 8 hours of MV in both groups (Table 1). During 8 hours of MV, 30% of the animals in the MV LPS group died (n =5: respectively, 4 hours, 5 hours and 5½ hours, and two mice died after 6 hours of MV). In the levosimendan-treated group, 12% (n =2) died before the end of the planned duration of MV (after 5 and 7½ hours of ventilation, respectively; *P* =0.2 between groups). Animals that did not survive until the end of the study were excluded from further biochemical analysis. Accordingly, 8 CON mice, 12 MV LPS mice and 14 MV LPS + L mice were included for biochemical analysis.

**Table 1:** **Arterial blood gas and alveolar-arterial gradient after 8 hours of mechanical ventilation and endotoxemia exposure** ^**a**^

	**pH**	**PaO**_**2**_ **(mmHg)**	**PaCO**_**2**_ **(mmHg)**	**HCO**_**3**_ **(mmol/L)**	**BE (mEq/L)**	**A-a gradient (mmHg)**
MV LPS + L	7.23 ± 0.0	153 ± 11	26 ± 1.3	11 ± 0.8	−16.6 ± 1.0	135 ± 9.4
MV LPS	7.24 ± 0.0	153 ± 14	25 ± 2.5	12 ± 1.0	−15.7 ± 1.3	136 ± 8.3

^a^Values are mean ± SEM. A-a, Alveolar-arterial; BE, Base excess; LPS, Lipopolysaccharide; MV LPS, Mechanically ventilated endotoxemic mice; MV LPS + L, Mechanically ventilated endotoxemic mice treated with levosimendan; PaCO_2_, Partial arterial carbon dioxide tension; PaO_2_, Partial arterial oxygen tension.

### Nitrosative and oxidative stress

**Figure 2:**
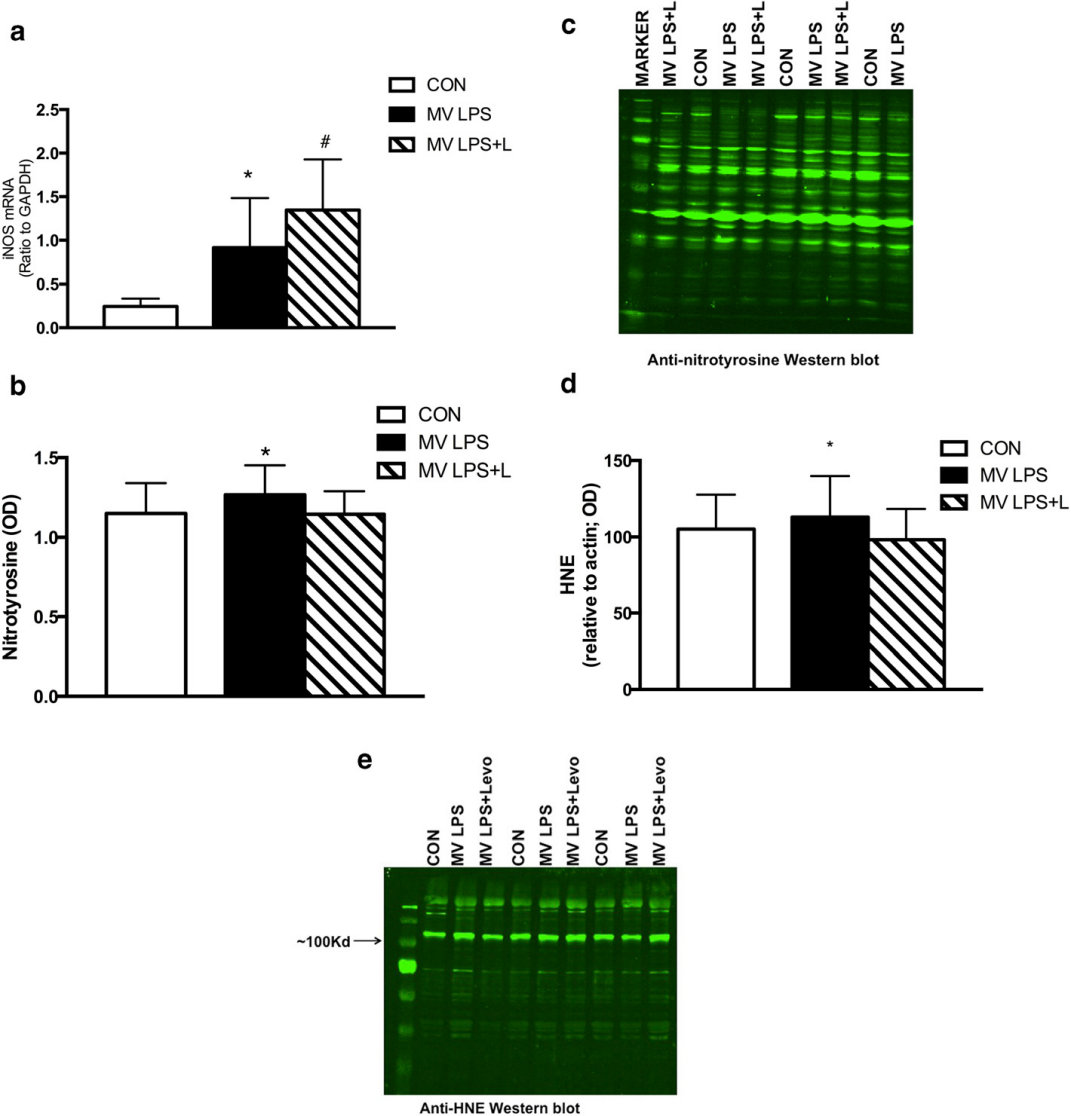
**Markers of nitrosative and oxidative stress. (A)** Expression of inducible nitric oxide synthase (iNOS) in the diaphragm. iNOS expression was increased in mechanically ventilated endotoxemic mice (MV LPS) versus unventilated mice (CON) (*P <0.05). iNOS expression was increased in mechanically ventilated endotoxemic mice treated with levosimendan (MV LPS + L) versus CON and MV LPS (#*P* <0.05). GAPDH, Glyceraldehyde 3-phosphate dehydrogenase; L, Levosimendan; LPS, Lipopolysaccharide. **(B)** Nitrotyrosine concentration in the diaphragm. Nitrotyrosine concentration was higher in MV LPS mice than in MV LPS + L mice (*P <0.05). OD, Optical density. **(C)** Representative nitrotyrosine blot. **(D)** 4-hydroxyl-2-nonenal (HNE) protein concentration relative to actin concentration in diaphragm. HNE protein concentration was higher in MV LPS than in MV LPS + L mice (*P <0.05). **(E)** Representative HNE protein blot.

To investigate the effects of levosimendan on nitrosative and oxidative stress in endotoxemic, mechanically ventilated mice, diaphragms were analyzed for iNOS expression, protein nitrosylation and HNE protein concentration. Diaphragm iNOS expression was significantly increased by 274% in LPS-exposed, ventilated mice (*P* <0.05 versus CON) (Figure 2A). Unexpectedly, levosimendan enhanced the expression of iNOS by 47% (Figure 2A) (*P* <0.05 for MV LPS versus MV LPS + L), but decreased diaphragm protein nitrosylation by 10% (Figure 2B,C) (*P* <0.05 for MV LPS versus MV LPS + L). Diaphragm HNE protein concentration was not significantly different between MV LPS and CON animals. Levosimendan decreased the amount of HNE proteins in the diaphragm by 13% compared with MV LPS (Figure 2D,E) (*P* <0.05 for MV LPS versus MV LPS + L).

### Inflammation

**Figure 3:**
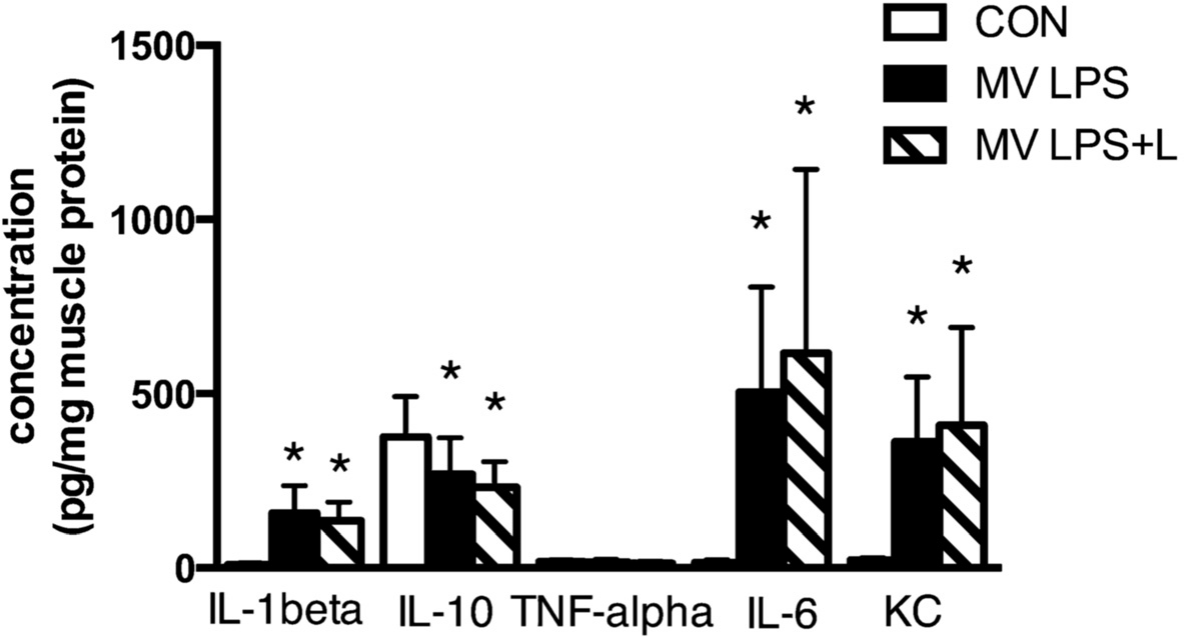
**Cytokine levels in diaphragm homogenates.** Mechanically ventilated endotoxemic mice (MV LPS) and mechanically ventilated endotoxemic mice treated with levosimendan (MV LPS + L) had increased levels of interleukin (IL)-1β, IL-6, keratinocyte-derived chemokine (KC) and decreased level of IL-10 (*P <0.05 versus unventilated mice (CON)). L, Levosimendan; LPS, Lipopolysaccharide.

**Figure 4:**
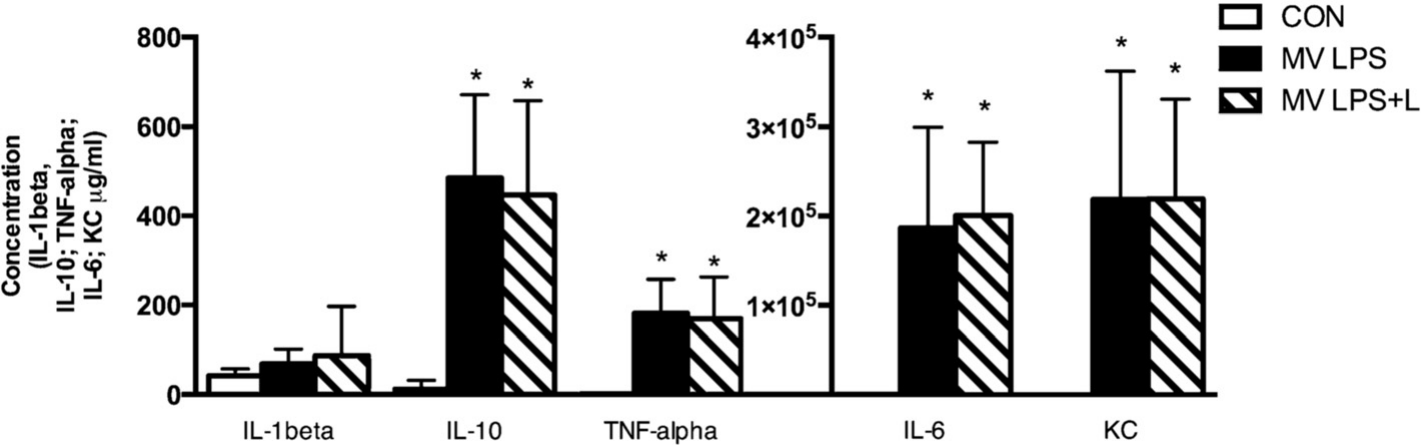
**Plasma cytokines.** Levels of interleukin (IL)-10, tumor necrosis factor (TNF)-α, IL-6 and keratinocyte-derived chemokine (KC) were increased in mechanically ventilated endotoxemic mice (MV LPS) and mechanically ventilated endotoxemic mice treated with levosimendan (MV LPS + L) (*P <0.05 versus unventilated mice (CON)). L = Levosimendan; LPS, Lipopolysaccharide.

In MV LPS mice, proinflammatory cytokines were significantly upregulated in diaphragm muscle and plasma (Figures 3 and 4). Levosimendan did not affect this inflammatory response.

### Caspase-3 and E3 ubiquitin ligases

Caspase-3 and the E3 ubiquitin ligases MuRF1 and MAFbx were analyzed as measures of muscle proteolysis. No differences in caspase-3 activity or MuRF1 mRNA were observed between groups. MAFbx expression was significantly increased in MV LPS and MV LPS + L animals compared with controls (ratios to GAPDH: 27 ± 12 for CON, 102 ± 35 for MV LPS and 114 ± 47 for MV LPS + L). Levosimendan did not affect expression of MAFbx in ventilated endotoxemic mice.

## Discussion

The main findings of the present study are that (1) levosimendan decreased protein nitrosylation and markers of oxidative stress in the diaphragm of endotoxemic mechanically ventilated mice, but that (2) levosimendan did not attenuate diaphragmatic and systemic inflammatory responses, diaphragmatic iNOS and E3 ubiquitin ligase expression or caspase-3 activity.

### Effects of endotoxemia and mechanical ventilation on the diaphragm

LPS in rodents is a widely used model to study the effects of endotoxemia on organ function, including respiratory muscles [28-31]. We found increased expression of iNOS in the diaphragm of these animals. This is in line with the only previous study in which researchers investigated a combination of endotoxemia and MV, where diaphragm weakness was associated with elevated levels of iNOS protein [10]. In addition, in the present study, proinflammatory cytokines were upregulated in the diaphragm of endotoxemic ventilated animals. This inflammatory response is consistent with earlier experimental models of either MV or endotoxemia [16,31,32]. MV and endotoxemia are known to increases proteolysis in the diaphragm, as supported by, for example, elevated caspase-3 activity and expression of the E3 ubiquitin ligases MuRF1 and MAFbx [5,33-35]. Expression of MAFbx was also enhanced in our endotoxemic ventilated mice. MuRF1 expression and caspase-3 activity were not elevated in endotoxemic ventilated mice. This suggests that the proteolytic response to a combination of MV and endotoxemia is weaker than that to MV or endotoxemia separately. Reduced proteolysis could preserve diaphragmatic function. This is supported by findings in a previous study in rats [10], where MV was shown to partly prevent the development of diaphragmatic dysfunction during endotoxemia. However, it should be acknowledged that, in the present study, the effect of MV solely on the diaphragm was not evaluated, as this was not required to test the hypothesis of the study.

It has been shown previously that endotoxemia is associated with pulmonary inflammation and elevates the A-a oxygen gradient [36]. In line with those observations, we found a high A-a gradient in endotoxemic ventilated mice (Table 1). The A-a gradient was not assessed in control mice in the present study. We have previously shown that the A-a oxygen gradient in healthy mice ventilated for 8 hours was higher than normal (79 ± 21 mmHg) [16], but it was significantly lower than reported in the present study for LPS-exposed ventilated mice. This indicates that systemic effects of LPS further impaired oxygen uptake in the endotoxemic ventilated groups of the present study.

### Effects of levosimendan

**Figure 5:**
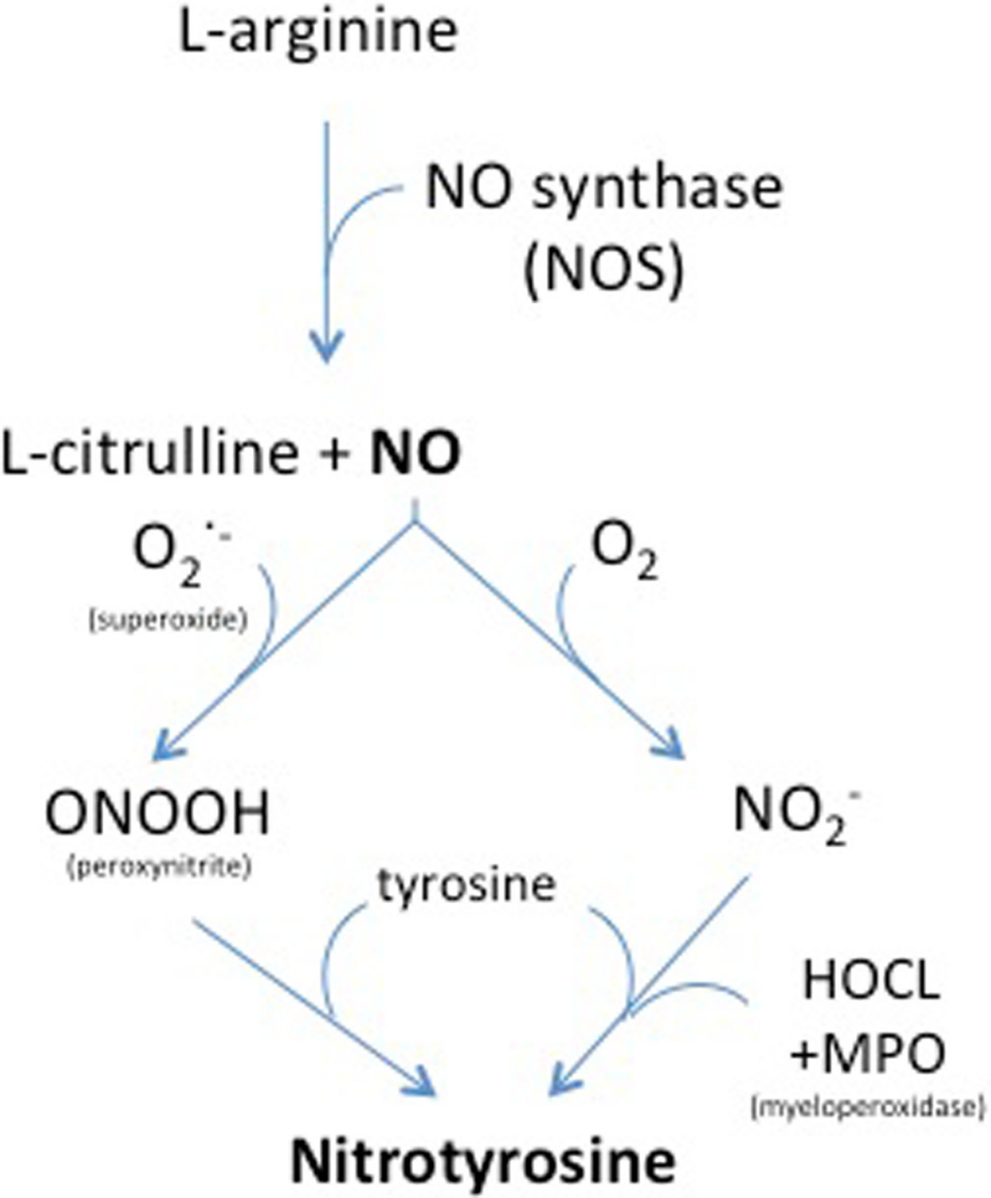
**Chemical reactions involved in the formation of nitrotyrosine.**

Among its inotropic effects through calcium sensitization, levosimendan has been shown to affect several intracellular pathways involved in oxidative stress and inflammation [21,23]. In the present study, levosimendan infusion decreased nitrosylated protein levels in ventilated endotoxemic mice (Figure 2B). Peroxynitrite formation is one of the chemical reactions involved in the nitrosylation of tyrosine residues in proteins (Figure 5) [37,38]. Peroxynitrite is an extremely reactive free radical generated from the reaction between nitric oxide and superoxide [39]. Nitric oxide is synthesized from l-arginine under the influence of the three NOS enzymes: iNOS, endothelial NOS and neuronal NOS [40].

In the present study, levosimendan did not reduce iNOS expression (Figure 2A) in the diaphragm of mechanically ventilated endotoxemic animals. This may suggest that levosimendan reduced peroxynitrite formation by lowering superoxide levels. Decreased superoxide levels are expected to be accompanied by a reduction of markers for oxidative stress. In line with that assumption, HNE protein levels (a marker for oxidative stress) were decreased in the diaphragm of levosimendan-treated mice (Figure 2D). In accordance with the possible ability of levosimendan to reduce oxidative stress, a recent investigation showed that levosimendan treatment increased protective antioxidant enzyme levels in renal tissues of rats [41]. Myeloperoxidases can induce tyrosine nitrosylation independently of peroxynitrite formation (Figure 5) [42]. Interestingly, previous experimental studies have shown that levosimendan reduces myeloperoxidase activity in the heart and spinal cord [43,44], but this has not been investigated in the diaphragm. Although we did not specifically investigate this pathway, we cannot exclude the possibility that levosimendan decreased protein nitrosylation by reducing myeloperoxidase activity.

In the present study, levosimendan did not attenuate the proinflammatory response to endotoxemia and MV in either plasma or the diaphragm (Figures 3 and 4). The lack of effect of levosimendan on plasma inflammation after levosimendan exposure is in line with previous studies [45,46]. In apparent contrast, in experimental sepsis induced by cecal ligation and incision, levosimendan did reduce high plasma levels of IL-1β [24]. However, in our animal model, plasma levels of IL-1β were not elevated after LPS exposure. Furthermore, levosimendan did not dampen MAFbx expression in endotoxemic ventilated mice. Interestingly, IL-6 is a well-known regulator of MAFbx gene expression [47]. The absence of an effect of levosimendan on plasma and diaphragmatic IL-6 levels could provide an explanation for the absence of a dampened effect of levosimendan on MAFbx expression. The activation of caspase-3, another essential player in muscle protein degradation, was reduced in the spleen of septic animals treated with levosimendan [24]. Also, in cardiac muscle cells, levosimendan could protect against hydrogen peroxide–induced caspase-3 activation [23]. In the present study, caspase-3 activity in the diaphragm was unchanged by levosimendan, but was not enhanced by endotoxemia and MV.

### Study limitations

The present study has limitations that should be acknowledged. First, the duration of MV and endotoxemia in the present study was relatively shorter than is usually the case in critically ill patients. It should be noted that MV in mice is challenging because of limited possibilities of vital function monitoring and the fact that, owing to very low intravascular volume (approximately 2 ml), no repetitive samples can be withdrawn for blood gas analysis. Nevertheless, in the present study, 8 hours of MV resulted in modulation of important biochemical pathways in the diaphragm, in line with the results of our previous study [16]. Therefore, this time period is appropriate for study of the effects of levosimendan on activation of inflammatory and proteolytic pathways. A second limitation of our study is that we did not assess the effects of levosimendan on diaphragm muscle function. Previously, we have shown that levosimendan improves diaphragm muscle function, both *in vitro* and *in vivo*, in healthy subjects, in patients with COPD and in animal models of congestive heart failure [18,19,48]. We can only speculate that it also will improve diaphragm function in the current model, but this should be confirmed in future studies. Third, as MV of endotoxemic mice is technically challenging, we were reluctant to invasively monitor cardiac output or tissue perfusion, which is of potential interest because levosimendan has been shown to both improve cardiac output and induce vasodilation [20]. Fourth, in the present study, LPS was used to induce an inflammatory response, and LPS administration to animals has been proven to be a predictable model of systemic inflammation [49]. However, it should be acknowledged that this is a model of systemic inflammation and does not necessarily reflect the physiological processes observed in human septic shock.

## Conclusions

In an animal model of endotoxemia and MV, levosimendan decreased markers of oxidative and nitrosative stress. Therefore, our data may suggest that the beneficial effects of levosimendan in clinical trials [20,50] may partly result from effects beyond calcium sensitization. The present study provides a rationale for investigating such a mechanism in a clinical study. In a current randomized clinical trial (ClinicalTrials.gov identifier NCT01721434), we are investigating levosimendan in patients difficult to wean from the ventilator.

## Key messages

- Levosimendan dampens nitrosative stress in the diaphragm of mechanically ventilated endotoxemic mice.
- Levosimendan did not attenuate diaphragmatic inflammation following 8 hours of MV in endotoxemic mice.

## Abbreviations

A-a: Alveolar-arterial
ANOVA: Analysis of variance
BE: Base excess
CON: Unventilated mice
COPD: Chronic obstructive pulmonary disease
DTT: Dithiothreitol
EGTA: Ethylene glycol tetraacetic acid
GAPDH: Glyceraldehyde 3-phosphate dehydrogenase
HNE: 4-Hydroxyl-2-nonenal protein
ICU: Intensive care unit
IL: Interleukin
iNOS: inducible nitric oxide synthase
KC: Keratinocyte-derived chemokine
KMA: Ketamine, medetomidine and atropine
LPS: Lipopolysaccharide
MAFbx: Muscle atrophy factor box
MuRF1: Muscle RING finger 1
MV: Mechanical ventilation
MV LPS: Mechanically ventilated endotoxemic mice
MV LPS + L: Mechanically ventilated endotoxemic mice treated with levosimendan
OD: Optical density
PaCO_2_: Partial arterial carbon dioxide tension
PaO_2_: Partial arterial oxygen tension
TNF-α: tumor necrosis factor-α

## References

[1] Levine S, Nguyen T, Taylor N, Friscia ME, Budak MT, Rothenberg P (2008). Rapid disuse atrophy of diaphragm fibers in mechanically ventilated humans. N Engl J Med.

[2] Jaber S, Petrof BJ, Jung B, Chanques G, Berthet JP, Rabuel C (2011). Rapidly progressive diaphragmatic weakness and injury during mechanical ventilation in humans. Am J Respir Crit Care Med.

[3] Callahan LA, Supinski GS (2009). Sepsis-induced myopathy. Crit Care Med.

[4] Hooijman PE, Beishuizen A, de Waard MC, de Man FS, Vermeijden JW, Steenvoorde P (2014). Diaphragm fiber strength is reduced in critically ill patients and restored by a troponin activator. Am J Respir Crit Care Med.

[5] Supinski GS, Ji X, Wang W, Callahan LA (2007). The extrinsic caspase pathway modulates endotoxin-induced diaphragm contractile dysfunction. J Appl Physiol.

[6] Hermans G, Agten A, Testelmans D, Decramer M, Gayan-Ramirez G (2010). Increased duration of mechanical ventilation is associated with decreased diaphragmatic force: a prospective observational study. Crit Care.

[7] Petrof B, Jaber S, Matecki S (2009). Ventilator-induced diaphragmatic dysfunction. Curr Opin Crit Care.

[8] Powers SK (2009). Calpain and caspase-3 are required for sepsis-induced diaphragmatic weakness. J Appl Physiol.

[9] Supinski GS, Vanags J, Callahan LA (2009). Effect of proteasome inhibitors on endotoxin-induced diaphragm dysfunction. Am J Physiol Lung Cell Mol Physiol.

[10] Ebihara S, Hussain SNA, Danialou G, Cho WK, Gottfried SB, Petrof BJ (2002). Mechanical ventilation protects against diaphragm injury in sepsis: interaction of oxidative and mechanical stresses. Am J Respir Crit Care Med.

[11] Betters JL, Criswell DS, Shanely RA, Van Gammeren D, Falk D, Deruisseau KC (2004). Trolox attenuates mechanical ventilation-induced diaphragmatic dysfunction and proteolysis. Am J Respir Crit Care Med.

[12] DeRuisseau KC, Kavazis AN, Deering MA, Falk DJ, Van Gammeren D, Yimlamai T (2005). Mechanical ventilation induces alterations of the ubiquitin-proteasome pathway in the diaphragm. J Appl Physiol.

[13] Levine S, Biswas C, Dierov J, Barsotti R, Shrager JB, Nguyen T (2011). Increased proteolysis, myosin depletion, and atrophic AKT-FOXO signaling in human diaphragm disuse. Am J Respir Crit Care Med.

[14] Vary TC (1998). Regulation of skeletal muscle protein turnover during sepsis. Curr Opin Clin Nutr Metab Care.

[15] van Hees HWH, Schellekens WJM, Linkels M, Leenders F, Zoll J, Donders R (2011). Plasma from septic shock patients induces loss of muscle protein. Crit Care.

[16] Schellekens WJM, van Hees HWH, Vaneker M, Linkels M, Dekhuijzen PNR, Scheffer GJ (2012). Toll-like receptor 4 signaling in ventilator-induced diaphragm atrophy. Anesthesiology.

[17] Schellekens WJM, van Hees HW, Kox M, Linkels M, Acuña GL, Dekhuijzen PNR (2014). Hypercapnia attenuates ventilator-induced diaphragm atrophy and modulates dysfunction. Crit Care.

[18] van Hees HWH, Dekhuijzen PNR, Heunks LMA (2009). Levosimendan enhances force generation of diaphragm muscle from patients with chronic obstructive pulmonary disease. Am J Respir Crit Care Med.

[19] Doorduin J, Sinderby CA, Beck J, Stegeman DF, van Hees HW, van der Hoeven JG (2011). The calcium sensitizer levosimendan improves human diaphragm function. Am J Respir Crit Care Med.

[20] Follath F, Cleland JGF, Just H, Papp JGY, Scholz H, Peuhkurinen K (2002). Efficacy and safety of intravenous levosimendan compared with dobutamine in severe low-output heart failure (the LIDO study): a randomised double-blind trial. Lancet.

[21] Sareila O, Korhonen R, Auvinen H, Hämäläinen M, Kankaanranta H, Nissinen E (2008). Effects of levo- and dextrosimendan on NF-κB-mediated transcription, iNOS expression and NO production in response to inflammatory stimuli. Br J Pharmacol.

[22] Boost KA, Hoegl S, Dolfen A, Czerwonka H, Scheiermann P, Zwissler B (2008). Inhaled levosimendan reduces mortality and release of proinflammatory mediators in a rat model of experimental ventilator-induced lung injury. Crit Care Med.

[23] Uberti F, Caimmi PP, Molinari C, Mary D, Vacca G, Grossini E (2011). Levosimendan modulates programmed forms of cell death through K_ATP_ channels and nitric oxide. J Cardiovasc Pharmacol.

[24] Scheiermann P, Ahluwalia D, Hoegl S, Dolfen A, Revermann M, Zwissler B (2009). Effects of intravenous and inhaled levosimendan in severe rodent sepsis. Intensive Care Med.

[25] Ottenheijm CAC, Heunks LMA, Geraedts MCP, Dekhuijzen PNR (2006). Hypoxia-induced skeletal muscle fiber dysfunction: role for reactive nitrogen species. Am J Physiol Lung Cell Mol Physiol.

[26] Ottenheijm CAC, Heunks LMA, Li YP, Jin B, Minnaard R, van Hees HWH (2006). Activation of the ubiquitin–proteasome pathway in the diaphragm in chronic obstructive pulmonary disease. Am J Respir Crit Care Med.

[27] Vaneker M, Joosten LA, Heunks LMA, Snijdelaar DG, Halbertsma FJ, van Egmond J (2008). Low-tidal-volume mechanical ventilation induces a Toll-like receptor 4-dependent inflammatory response in healthy mice. Anesthesiology.

[28] Supinski GS, Callahan LA (2006). Caspase activation contributes to endotoxin-induced diaphragm weakness. J Appl Physiol.

[29] Hussain SN, Giaid A, El Dawiri Q, Sakkal D, Hattori R, Guo Y (1997). Expression of nitric oxide synthases and GTP cyclohydrolase I in the ventilatory and limb muscles during endotoxemia. Am J Respir Cell Mol Biol.

[30] Boczkowski J, Lisdero CL, Lanone S, Samb A, Carreras MC, Boveris A (1999). Endogenous peroxynitrite mediates mitochondrial dysfunction in rat diaphragm during endotoxemia. FASEB J.

[31] Demoule A, Divangahi M, Yahiaoui L, Danialou G, Gvozdic D, Labbe K (2006). Endotoxin triggers nuclear factor-κB-dependent up-regulation of multiple proinflammatory genes in the diaphragm. Am J Respir Crit Care Med.

[32] Borge BAS, Kalland KH, Olsen S, Bletsa A, Berggreen E, Wiig H (2009). Cytokines are produced locally by myocytes in rat skeletal muscle during endotoxemia. Am J Physiol Heart Circ Physiol.

[33] McClung JM, Kavazis AN, DeRuisseau KC, Falk DJ, Deering MA, Lee Y (2007). Caspase-3 regulation of diaphragm myonuclear domain during mechanical ventilation-induced atrophy. Am J Respir Crit Care Med.

[34] McClung JM, Kavazis AN, Whidden MA, Deruisseau KC, Falk DJ, Criswell DS (2007). Antioxidant administration attenuates mechanical ventilation-induced rat diaphragm muscle atrophy independent of protein kinase B (PKB Akt) signalling. J Physiol.

[35] Fareed MU, Evenson AR, Wei W, Menconi M, Poylin V, Petkova V (2006). Treatment of rats with calpain inhibitors prevents sepsis-induced muscle proteolysis independent of atrogin-1/MAFbx and MuRF1 expression. Am J Physiol Regul Integr Comp Physiol.

[36] Matute-Bello G, Frevert CW, Martin TR (2008). Animal models of acute lung injury. Am J Physiol Lung Cell Mol Physiol.

[37] Supinski G, Stofan D, Callahan LA, Nethery D, Nosek TM, DiMarco A (1999). Peroxynitrite induces contractile dysfunction and lipid peroxidation in the diaphragm. J Appl Physiol.

[38] Pacher P, Beckman JS, Liaudet L (2007). Nitric oxide and peroxynitrite in health and disease. Physiol Rev.

[39] Beckman JS, Koppenol WH (1996). Nitric oxide, superoxide, and peroxynitrite: the good, the bad, and ugly. Am J Physiol.

[40] Vallance P, Leiper J (2002). Blocking NO synthesis: how, where and why?. Nat Rev Drug Discov.

[41] Gecit I, Kavak S, Yüksel MB, Basel H, Bektas H, Gümrükçüoglu HA (2014). Effect of short-term treatment with levosimendan on oxidative stress in renal tissues of rats. Toxicol Ind Health.

[42] van der Vliet A, Eiserich JP, Halliwell B, Cross CE (1997). Formation of reactive nitrogen species during peroxidase-catalyzed oxidation of nitrite: a potential additional mechanism of nitric oxide-dependent toxicity. J Biol Chem.

[43] Yapici D, Altunkan Z, Ozeren M, Bilgin E, Balli E, Tamer L (2008). Effects of levosimendan on myocardial ischaemia-reperfusion injury. Eur J Anaesthesiol.

[44] Katircioglu SF, Seren M, Parlar AI, Turan NN, Manavbasi Y, Aydog G (2008). Levosimendan effect on spinal cord ischemia-reperfusion injury following aortic clamping. J Card Surg.

[45] Chew MS, Hawthorne WJ, Bendall J, Whereat S, Huang S, Ting I (2011). No beneficial effects of levosimendan in acute porcine endotoxaemia. Acta Anaesthesiol Scand.

[46] Zager RA, Johnson AC, Lund S, Hanson SY, Abrass CK (2006). Levosimendan protects against experimental endotoxemic acute renal failure. Am J Physiol Renal Physiol.

[47] Baltgalvis KA, Berger FG, Peña MMO, Davis JM, White JP, Carson JA (2009). Muscle wasting and interleukin-6-induced atrogin-I expression in the cachectic *Apc*^*Min/+*^ mouse. Pflugers Arch.

[48] van Hees HWH, Andrade Acuña G, Linkels M, Dekhuijzen PNR, Heunks LMA (2011). Levosimendan improves calcium sensitivity of diaphragm muscle fibres from a rat model of heart failure. Br J Pharmacol.

[49] Remick DG, Ward PA (2005). Evaluation of endotoxin models for the study of sepsis. Shock.

[50] Fuhrmann JT, Schmeisser A, Schulze MR, Wunderlich C, Schoen SP, Rauwolf T (2008). Levosimendan is superior to enoximone in refractory cardiogenic shock complicating acute myocardial infarction. Crit Care Med.

